# Preformed mincle dimers stabilized by an interchain disulfide bond in the neck region

**DOI:** 10.1093/glycob/cwae083

**Published:** 2024-10-03

**Authors:** Yu Liu, Kurt Drickamer, Maureen E Taylor

**Affiliations:** Department of Life Sciences, Sir Ernst Chain Building, Imperial College London, Exhibition Road, London SW7 2AZ, United Kingdom; Department of Life Sciences, Sir Ernst Chain Building, Imperial College London, Exhibition Road, London SW7 2AZ, United Kingdom; Department of Life Sciences, Sir Ernst Chain Building, Imperial College London, Exhibition Road, London SW7 2AZ, United Kingdom

**Keywords:** carbohydrate-binding protein, cell signaling, glycan-binding receptor, Innate immunity, Lectin

## Abstract

The sugar-binding receptor mincle stimulates macrophages when it encounters surface glycans on pathogens, such as trehalose dimycolate glycolipid in the outer membrane of mycobacteria. Binding of oligosaccharide ligands to the extracellular C-type carbohydrate-recognition domain (CRD) in mincle initiates intracellular signaling through the common Fc receptor γ (FcRγ) adapter molecule associated with mincle. One potential mechanism for initiation of signaling involves clustering of receptors, so it is important to understand the oligomeric state of mincle. Affinity purification of mincle from transfected mammalian cells has been used to show that mincle exists as a pre-formed, disulfide-linked dimer. Deletion of cysteine residues and chemical crosslinking further demonstrate that the dimers of mincle are stabilized by a disulfide bond between cysteine residues in the neck sequence that links the CRD to the membrane. In contrast, cysteine residues in the transmembrane region of mincle are not required for dimer formation or association with FcRγ. A protocol has been developed for efficient production of a disulfide-linked extracellular domain fragment of mincle in a bacterial expression system by appending synthetic dimerization domains to guide dimer formation in the absence of the membrane anchor.

## Introduction

Mincle, the macrophage-inducible C-type lectin also known as CLEC-4E, is expressed on the surface of macrophages and interacts with pathogenic mycobacterial species such as *Mycobacterium tuberculosis* by binding to trehalose dimycolate glycolipid in the mycobacterial outer membrane (Matsumoto et al. 1999; Ishikawa et al. 2009; Matsunaga and Moody 2009; Schoenen et al. 2010; Lang 2013). The carbohydrate-recognition domain (CRD) of mincle binds to both the trehalose headgroup and the hydrophobic portions of the glycolipid (Feinberg et al. 2013). Mincle also binds fungal glycolipids and apoptotic cells (Wells et al. 2008; Yamasaki et al. 2008; Drummond et al. 2011; Ishikawa et al. 2013). Ligand binding to mincle upregulates production of nitric oxide and pro-inflammatory cytokines IL-6 and TNFα (Ishikawa et al. 2009).

Because mincle lacks intracellular signaling motifs, association with the ITAM-bearing common Fc receptor γ (FcRγ) adapter molecule is required for signal initiation. Binding of ligands such as trehalose dimycolate to mincle associated with FcRγ initiates a pro-inflammatory signaling pathway through Syk kinase and CARD9 (Yamasaki et al. 2008; Werninghaus et al. 2009). Mutagenesis studies suggest that association of mincle with FcRγ is mediated by electrostatic interaction between a positively charged arginine in the transmembrane portion of mincle and a negatively charged aspartate in the FcRγ adapter molecule (Yamasaki et al. 2008). This mechanism of interaction is similar to other receptors that activate the FcRγ, DAP10 and DAP12 adapters (Call and Wucherpfennig 2007; Lanier 2009). One potential mechanism for initiating signaling involves clustering of mincle molecules at the cell surface so it is important to understand the oligomeric state of mincle.

Mincle is structurally related to several other membrane receptors, including blood dendritic cell antigen 2 (BDCA-2) and dectin-2 as well as macrophage C-type lectin (Kerscher et al. 2013). Each of these receptors consists of a short cytoplasmic N-terminal domain, a hydrophobic transmembrane sequence and an extracellular neck linking to a C-terminal C-type CRD. Attempts to study mincle organization on the cell surface suggest that disulfide-bonded heterodimers of mincle and macrophage C-type lectin can be formed in human embryonic kidney (HEK) cells co-transfected with both receptors (Lobato-Pascual et al. 2013). However, in those studies, the oligomeric state of mincle in the absence of macrophage C-type lectin was not investigated.

In the studies reported here, an affinity purification method has been used to demonstrate that mincle in transfected cells forms disulfide-linked dimers that are linked through a cysteine residue in the neck region. Understanding how mincle oligomerizes on the cell surface provides a model to be compared with related receptors, such as BDCA-2 and dectin-2. It also helps to build a picture of how the ligand-receptor interaction triggers immune signaling.

## Results

### Expression of mincle in HEK cells

**Fig. 1:**
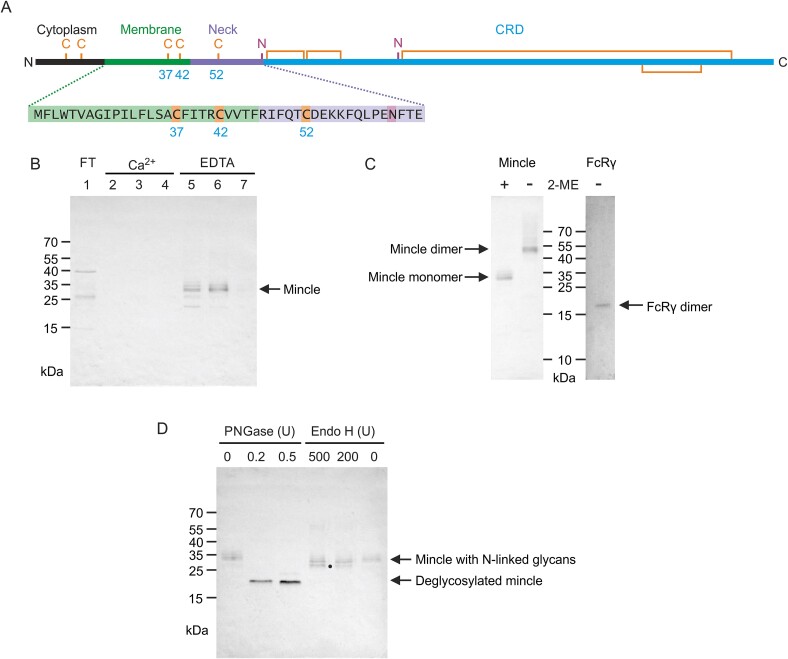
Characterization of mincle expressed in HEK cells. Mincle was analyzed by SDS-polyacrylamide gel electrophoresis and western blotting with anti-mincle and anti-FcRγ antibodies. A) Domain structure of mincle, highlighting the positions of cysteine residues (denoted C) in the overall sequence and specifically in the transmembrane (green) and neck (purple) regions. Asparagine residues (denoted N) that are sites of potential N-linked glycosylation are also indicated. B) Affinity purification of mincle from transfected HEK cells on trehalose-Sepharose. Samples were prepared in the presence of 2-mercaptoethanol. C) Demonstration of disulfide-linked mincle dimer formation and co-purification of mincle and FcRγ adapter. Samples were analyzed in the absence or presence of 2-mercaptoethanol (2-ME). D) Results of endoglycosidase treatment of mincle. Samples were prepared in the presence of 2-mercaptoethanol. Dot highlights partial reduction in molecular weight following endoglycosidase H digestion.

Mincle was affinity purified from detergent-solubilized cell extracts from HEK cells doubly transfected with plasmids encoding full length human mincle (Fig. 1A) and FcRγ. Taking advantage of the Ca^2+^-dependence of the C-type CRD, mincle bound to a trehalose-Sepharose column in the presence of Ca^2+^ was eluted with EDTA-containing buffer (Fig. 1B). Analysis in the presence and absence of reducing agent showed that the mincle polypeptide exists as a disulfide-linked dimer and probing of a parallel western blot confirmed that FcRγ co-elutes with mincle and also runs as a disulfide-linked dimer (Fig. 1C), which is formed through a cysteine residue in the short extracellular domain (Deng et al. 2024). There is no evidence on the non-reducing gels that mincle and FcRγ subunits are linked to each other by disulfide bonds.

Endoglycosidases were used to provide an indication of the localization of the mincle dimer within cells, by monitoring the state of processing of attached N-linked glycans (Fig. 1D). As expected, the N-linked glycans were removed upon treatment with PNGase, resulting in a mobility shift consistent with loss of two glycans. In contrast, there was very little endoglycosidase H-sensitive glycoprotein, which would correspond to material in the early secretory pathway that bears high mannose oligosaccharides. Partial reduction in the molecular weight of some of the endoglycosidase H-treated material may reflect the presence of a mixture of high mannose and processed complex glycans attached to some of the polypeptides. These results indicate that all of the mincle has been glycosylated at both target asparagine residues and at least one of the attached glycans is processed. The mincle molecules are thus likely to have been transported through the Golgi.

### Characterization of mincle lacking cysteine in the extracellular neck region

The eight cysteine residues in the CRD of mincle form four intrachain disulfide bonds (Feinberg et al. 2016). However, there are three other cysteine residues in the mincle sequence that could form interchain disulfide bonds: two in the transmembrane domain and one in the neck (Fig. 1A). Although it has been reported that intramembrane cysteine residues are capable of forming interchain disulfide bonds, such as in the ζζ dimer in the T cell receptor complex (Rutledge et al. 1992), the role of the neck cysteine residue (Cys52) in mincle dimerization was investigated initially because it is exposed to a more oxidizing environment in the lumen of the endoplasmic reticulum.

**Fig. 2:**
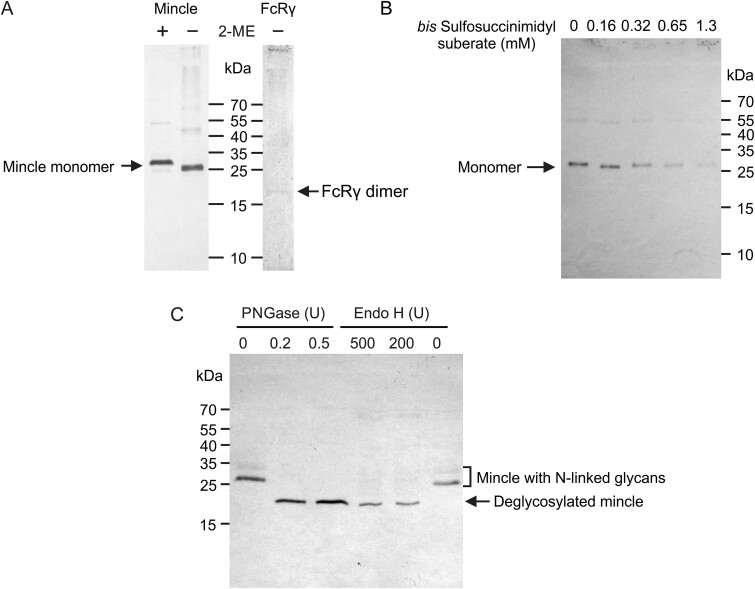
Characterization of mincle lacking cysteine residue in the neck region. Cys52Ala mincle purified by affinity chromatography on trehalose-Sepharose was analyzed by SDS-polyacrylamide gel electrophoresis and western blotting. Blots were developed with anti-mincle and anti-FcRγ antibodies. A) Analysis of mincle in the absence or presence of 2-mercaptoethanol and co-purification of FcRγ adapter. B) Crosslinking with *bis*-sulfosuccinimidyl suberate. Although reaction with the reagent does not result in dimer formation, extensive derivatization of lysine residues at higher reagent concentrations results in loss of reactivity with the anti-mincle antibody. C) Digestion with endoglycosidases.

To examine the role of the neck region cysteine in disulfide-linked dimer formation, HEK cells were co-transfected with a Cys52Ala mutant of mincle, along with FcRγ. The mutant form of mincle could be affinity purified following the procedure used for the wild type protein, but substitution of Cys52 abolishes disulfide-linked mincle dimer formation (Fig. 2A, left). Nevertheless, association with FcRγ dimer is still observed (Fig. 2A, right). Chemical crosslinking with the bifunctional reagent *bis*-sulfosuccinimidyl suberate did not result in trapping of mincle dimers (Fig. 2B). The results demonstrate that the neck cysteine residues in a mince dimer form an interchain disulfide bond that is essential for dimer stability.

Much of the purified Cys52Ala mutant mincle is susceptible to endoglycosidase H digestion, indicating that this mutant protein bears mostly high-mannose glycans (Fig. 2C). In the absence of formation of disulfide-linked dimers, the mincle is thus more likely to be retained in the endoplasmic reticulum instead of moving to the Golgi and the cell surface.

### Characterization of mincle lacking intramembrane cysteine residues

**Fig. 3:**
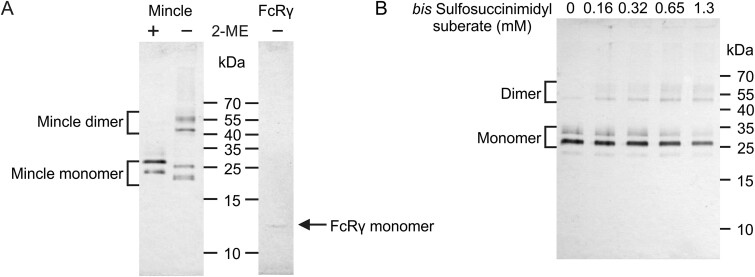
Characterization of mincle lacking cysteine residues in the membrane. Mincle purified by affinity chromatography on trehalose-Sepharose was analyzed by SDS-polyacrylamide gel electrophoresis and western blotting. Blots were developed with anti-mincle and anti-FcRγ antibodies. A) Mutant mincle analyzed in the absence or presence of 2-mercaptoethanol (2-ME) and copurification of FcRγ adapter. B) Crosslinking with *bis*-sulfosuccinimidyl suberate. Gel in B was run in the presence of 2-mercaptoethanol.

To determine if the intramembrane cysteine residues Cys37 and Cys42 also play a role in dimer formation, both of these residues were replaced with alanine. Mincle can be detected in cells transfected with this mutant mincle, along with FcRγ, but the protein appears as multiple bands on SDS-polyacrylamide gels (Fig. 3A). In this and subsequent experiments, the presence of double bands probably reflects increased sensitivity of this mutant protein to proteolysis. Nevertheless, in the absence of reducing agent, at least half of the protein appears as disulfide-linked dimers (Fig. 3A). Chemical crosslinking also confirmed the presence of dimer (Fig. 3B). As with the neck region cysteine mutant, copurification of FcRγ was still observed, but in this case the FcRγ runs as a monomer. The results suggest that the neck region cysteine of mincle is sufficient to support dimer formation, but the absence of the intramembrane cysteine residues results in inefficient assembly of the complex in the endoplasmic reticulum.

Investigation of wild type and mutant mincle in cells thus shows that mincle forms a disulfide-linked homodimer through the neck cysteine residue.

### Dimeric extracellular domain fragments of mincle

Bacterial systems for expression of the mincle dimeric extracellular domain could provide an important tool to facilitate further analysis of how the dimers are organized and how the CRDs are presented at the cell surface. Because mincle dimers are stabilized by an interchain disulfide bond in the neck region, formation of disulfide-bonded covalent dimers of the extracellular domain can be used to monitor expression of biologically relevant dimers.

Bacterial expression studies were conducted with the extracellular portion of cow mincle because the CRD from cow mincle has been well characterized structurally and is expressed efficiently in *E. coli* (Feinberg et al. 2013, 2016). The extracellular and transmembrane portions of cow and human mincle share a high degree of sequence identity and the positions of all of the cysteine residues in these regions are conserved. The CRDs also show very similar Ca^2+^- and pH-dependent sugar-binding properties and the structure of the CRD from cow mincle has been successfully used to model the human protein (Jégouzo et al. 2014). Thus, the orientation of CRDs in a cow mincle extracellular domain dimer will provide a model for how CRDs are arranged in the human ortholog.

**Fig. 4:**
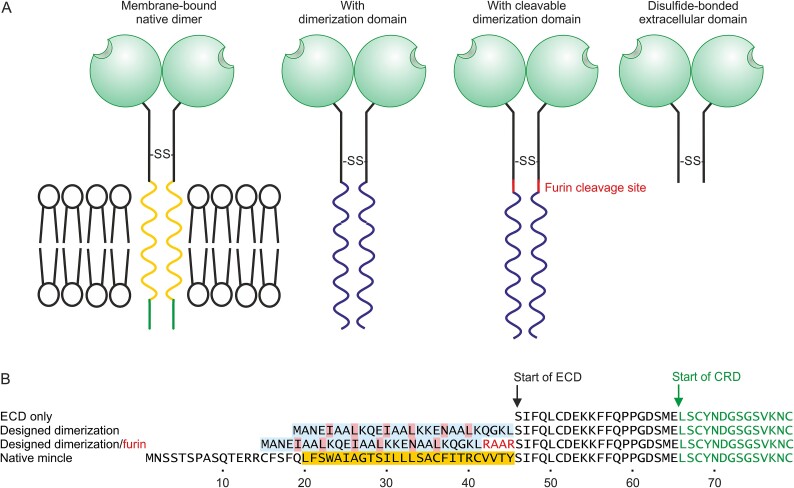
Strategy for producing dimeric mincle extracellular domain using dimerization domains. A) Organization of mincle constructs. B) Sequences of extracellular domain constructs for cow mincle. Transmembrane domain is highlighted in orange. Dimerization sequences are highlighted in blue, with pink shading of heptad repeat residues.

**Fig. 5:**
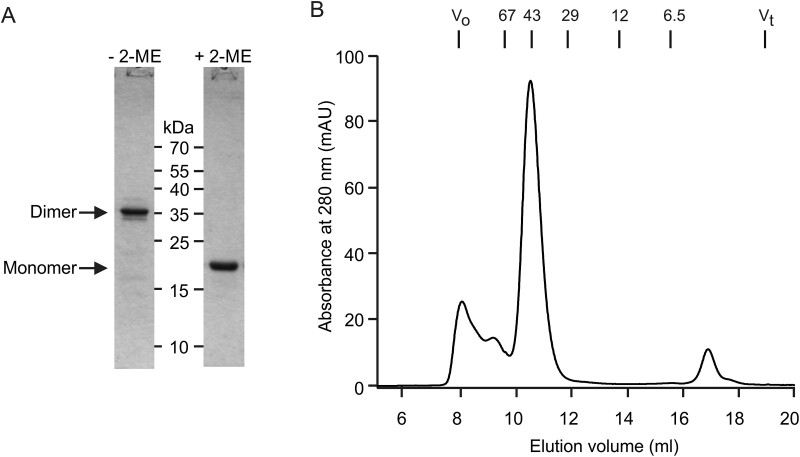
Analysis of affinity-purified mincle extracellular domain with synthetic 3-heptad dimerization domain. A) SDS-polyacrylamide gel electrophoresis in the presence and absence of 2-mercaptoethanol (2-ME). Gels were stained with Coomassie blue. B) Superdex S75 gel filtration analysis. Elution positions of standards are indicated in kDa at the top. The expected molecular weight of the dimeric protein is 44 kDa.

Initial attempts to express the extracellular domain of mincle in *E. coli* did not result in formation of dimers and attempts to induce dimer formation by catalyzing oxidation with Cu^2+^-o-phenanthroline (Steck 1972) were unsuccessful. As an alternative approach, dimerization domains were appended at the N-terminus of the extracellular domain, replacing the membrane anchor (Fig. 4). Two different domains that form dimeric coiled-coils of α-helices were explored: a modified version of the oligomerization domain from the transcription factor GCN4 and a designed amphipathic helical domain containing 3 heptad repeats (Harbury et al. 1993; Ciani et al. 2010; Fletcher et al. 2012). Both the GCN4 dimerization domain and the designed sequence facilitated formation of disulfide-linked dimers, but the version with the designed domain had better solubility characteristics. Analysis of this construct, purified by trehalose-Sepharose affinity chromatography, on non-reducing gels and by gel filtration confirmed that stable, covalent dimers were formed following renaturation (Fig. 5). A truncated version with 2 heptad repeats forms dimers less efficiently than the 3-heptad version (data not shown).

**Fig. 6:**
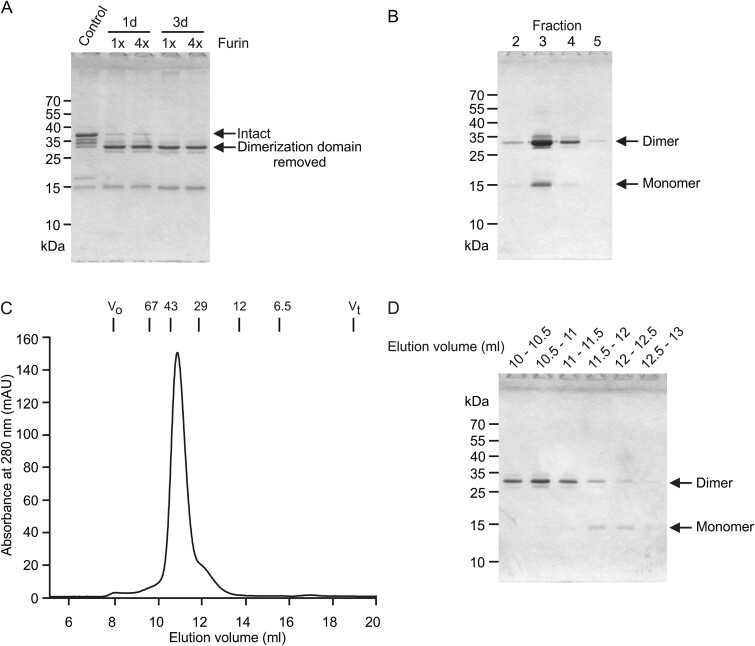
Preparation of disulfide-bonded mincle extracellular domain dimers. A) SDS-polyacrylamide gel showing test cleavage of dimerization domain from mincle extracellular domain. B) SDS-polyacrylamide gel of elution fractions from a 1-mL trehalose-Sepharose affinity column after furin cleavage. Mincle was eluted with EDTA in 0.5-mL fractions, aliquots of fractions were run on the gel in the absence of 2-mercaptoethanol, and gel was stained with Coomassie blue. C) Superdex S75 gel filtration fractionation of furin-cleaved mincle (D) SDS-polyacrylamide gel of fractions from Superdex S75 column. All gels were run in the absence of reducing agent.

Formation of the disulfide-linked dimer is directed by the dimerization domain, but once formed the disulfide bond should stabilize the dimer even in the absence of the fused domain. In order to facilitate removal of the dimerization domain after dimer formation, various protease cleavage site were introduced between the dimerization domain and the mincle extracellular domain. Protease digestion of constructs containing appropriate target sequences showed that furin cleavage was both specific and compatible with retention of interchain disulfide bonds. The presence of a furin cleavage site did not affect dimer formation and test digestions with furin showed that the dimer remained intact during extended digestion (Fig. 6A). The furin-cleaved dimer was successfully repurified on trehalose-Sepharose (Fig. 6B). Gel filtration chromatography resulted in a pure preparation of covalently-linked dimers of the expected molecular weight (Fig. 6C and D).

During repurification of the furin-treated protein on trehalose-Sepharose (Fig. 6B), comparing fractions 2 and 4 indicates that later-eluting material is enriched in the disulfide-linked dimer, probably reflecting increased avidity resulting from the presence of two binding sites in a single covalent dimer. This observation and the presence of a small amount of monomeric protein on the gel filtration column (Fig. 6D), suggest that in the absence of the disulfide bond, the dimer is not stable.

## Discussion

The work described here shows that mincle is expressed as a disulfide-bonded dimer formed by a single neck cysteine residue in each polypeptide. The presence of a disulfide bond in the neck domain confirms that mincle exists as a preformed dimer on cell surfaces.

The characteristics of the extracellular domain fragments of mincle provide some insights into the nature of the interactions that lead to dimer formation. The experiments employing the extracellular domain without a fused dimerization sequence suggest that interactions between the CRDs, which make up the major portion of the extracellular domain, are not sufficient to stabilize the dimer. The fact that the naturally occurring disulfide bond can be formed when the N-termini of the polypeptides have been brought into close proximity indicates that they are interacting in a native-like way in the extracellular domain construct. The development of a readily expressed dimeric form of the extracellular domain of mincle provides a potential starting point for structural analysis of the arrangement of the CRDs in the dimer.

The presence of the neck region disulfide bond distinguishes mincle from the structurally related family members BDCA-2 and dectin-2, which also exist as dimers but lack interchain disulfide bonds (Liu et al. 2024). However, a single-nucleotide polymorphism found in BDCA-2 results in replacement of a serine residue in the neck region by a cysteine residue that corresponds to the interchain disulfide bond-forming cysteine residue in the neck region of mincle. Characterization of this BDCA-2 variant confirms formation of a disulfide-linked dimer as seen with mincle (Liu et al. 2024). This result suggests that the neck interactions are likely to be similar in all of the receptors in spite of differences in the disulfide bond arrangement.

The neck region of human mincle also differs from dectin-2 and BDCA-2 by the presence of an N-glycosylation site (Fig. 1A). A glycan attached at this position could affect the conformation of the neck or potentially provide protection from proteolysis if the neck is in an extended conformation. There is no experimental evidence regarding the significance of this glycosylation site. However, in addition to being absent from dectin-2 and BDCA-2, the site is not conserved in cow or mouse mincle, making a structural role for the glycan less likely.

**Fig. 7:**
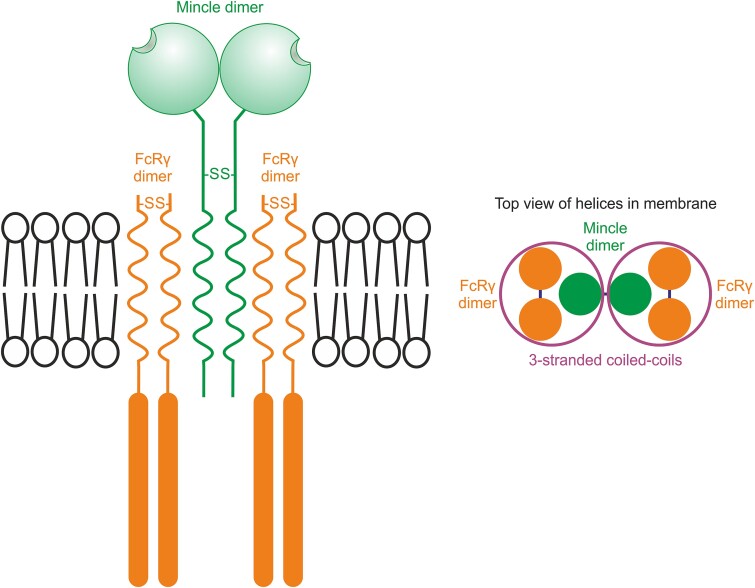
Model of a proposed complex between mincle dimer and FcRγ dimers. The model shows that a disulfide-linked FcRγ dimer interacts with a single receptor polypeptide to form a three-stranded coiled-coil of α helices (Deng et al. 2024) and as shown here the mincle part of the complex is a disulfide-linked dimer. The resulting hexameric complex is analogous to that proposed for the NKG2D receptor and the DAP12 signaling adapter (Garrity et al. 2005).

A proposed arrangement of the mincle-FcRγ complex is shown in Fig. 7. The model is informed by the evidence presented here that the mincle part of the complex is a disulfide-linked dimer, combined with published information on other receptors that interact with FcRγ and the related DAP10 and DAP12 adapters. In other FcRγ complexes, a disulfide-linked FcRγ dimer interacts with a single receptor polypeptide to form a three-stranded coiled-coil of α helices (Deng et al. 2024) and a similar three-stranded coil is formed by a single helix of NKG2C and a dimeric DAP12 adapter (Call et al. 2010). In analogous complexes of the NKG2D receptor and the DAP10 signaling adapter, an NKG2D receptor dimer interacts with two adapter dimers (Garrity et al. 2005).

A key point about the presence of the interchain disulfide bond in mincle is that it demonstrates that the mincle dimer is preformed even in the absence of a stimulating ligand, so dimerization cannot drive initiation of signaling. The dimeric arrangement of mincle is thus consistent with the alternative suggestion that initiation of signaling by glycan ligands involves crosslinking of preformed dimers into larger clusters. Mincle is a target for natural adjuvants, such as mycobacterial trehalose dimycolate, as well as synthetic analogs such as trehalose dibehentate (Ishikawa et al. 2009; Matsunaga and Moody 2009; Schoenen et al. 2010). Thus, further examination of the structure of extracellular domain dimers to define how the binding sites are arranged may provide a basis for further design of molecules that can crosslink mincle dimers and thus serve as improved adjuvants (Decout et al. 2017).

## Materials and methods

### Co-expression of mincle and FcRγ in HEK cells

DNA coding for human mincle was cloned into expression vector pSF-CMV-EMCV-Zeo (Oxford Genetics) and DNA coding for human FcRγ was cloned into vector pIRESHyg2 (Clontech). HEK 293 cells were transfected with 1.25 μg of each plasmid using lipofectamine 3000 reagent (Invitrogen) following the manufacturer’s protocol. After 48 h, cells were moved to medium containing 300 μg/mL zeocin and 200 μg/mL hygromycin (Invitrogen). Single colonies of zeocin- and hygromycin-resistant cells were picked and transferred to a 24-well plate before being passaged into 25 cm^2^ or 75 cm^2^ flasks. Cell lines expressing FcRγ and mincle with either the single neck cysteine residue mutated to alanine (Cys52Ala) or with both of the transmembrane cysteine residues replaced by alanine (Cys37Ala and Cys42Ala) were generated in the same way. Cysteine mutations were incorporated using synthetic oligonucleotides.

For protein isolation, cells from two 75-cm^2^ flasks were rinsed twice with phosphate-buffered saline, harvested and lysed by homogenization in a 1-mL syringe in 1 mL of cold lysis buffer: 0.15 M NaCl, 25 mM Tris pH 7.8, 2.5 mM CaCl_2_, and 1% Triton X-100, containing 10 μL of protease inhibitors (Merck Millipore), and 0.5 μL of Benzonase (Sigma). Following centrifugation at 16,000 × g for 10 min at 4 °C, mincle was purified on a 2-mL trehalose-Sepharose column (Fornstedt and Porath 1975). After application of the sample, the column was washed with 3.5 mL of 0.15 M NaCl, 25 mM Tris pH 7.8, 2.5 mM CaCl_2_, and 0.1% Triton X-100 and eluted with 3.5 mL of 0.15 M NaCl, 25 mM Tris pH 7.8, 2.5 mM EDTA and 0.1% Triton X-100. Protein was precipitated by addition of trichloroacetic acid (TCA) to 10%, incubation for 10 min at 4 °C and centrifugation at 16,000 g for 5 min at room temperature. Following two washes with 0.5 mL of ethanol:ether (1:1), pellets were dried and dissolved in SDS-polyacrylamide gel sample buffer with or without 1% 2-mercaptoethanol by heating for 5 min at 100 °C. In most experiments, protein eluted in the final 3 mL, corresponding to Fraction 5, 6 and 7 in Fig. 1B, was pooled.

### PNGase F / Endo H treatment

Affinity purified mincle was TCA precipitated, resuspended in 0.5% SDS containing 40 mM dithiothreitol, and heated at 100 °C for 10 min. Aliquots were incubated either with PNGase F (Roche) in 50 mM sodium phosphate pH 7.5 and 1% NP-40 at 37 °C for 15 min or endoglycosidase H_f_ (New England Biolabs) in 50 mM sodium acetate pH 6 at 37 °C for 15 min. Digestions were stopped by addition of 2x SDS-gel sample buffer and heating at 100 °C for 5 min.

### Chemical crosslinking

Lysate of HEK transfectants expressing mincle and FcRγ was fractionated by affinity chromatography on a 1-mL trehalose-Sepharose column, which was rinsed with 5 mL of wash buffer (0.15 M NaCl, 25 mM HEPES pH 7.8, 2.5 mM CaCl_2_, and 0.1% Triton X-100) and eluted in five 0.5-mL fractions with elution buffer (0.15 M NaCl, 25 mM HEPES pH 7.8, 2.5 mM EDTA and 0.1% Triton X-100). Crosslinking reagent *bis*(sulfosuccinimidyl) suberate (Pierce) was incubated with affinity-purified mincle at room temperature for 30 min, followed by TCA precipitation for gel analysis.

### Expression and purification of extracellular domain fragments

Synthetic oligonucleotides coding for a 3-heptad version of a dimerization domain based on the sequence designated a GCN4 dimerization domain (Harbury et al. 1993; Ciani et al. 2010) or CC-pIL-I17N (Fletcher et al. 2012) were appended to DNA coding for the extracellular regions of bovine mincle. Fusion proteins were expressed from pT5T plasmids (Eisenberg et al. 1990) in *Escherichia coli* strain BL21(DE3), renatured and purified as previously described (Feinberg et al. 2013) except that guanidine-solubilized protein was diluted slowly into 4 volumes of 0.5 M NaCl, 25 mM Tris-Cl 7.8, and 25 mM CaCl_2_ before dialysis and affinity purification on trehalose-Sepharose.

### Furin cleavage

Furin digestions were performed in 150 mM NaCl, 25 mM Tris-Cl, pH 7.8, 0.5% Triton X-100, 5 mM CaCl_2_. For test digestions, aliquots of approximately 2.5 μg of mincle in 25 μL were digested with 1–4 units of furin (New England Biolabs) at 37 °C. For preparative digestion, approximately 500 μg of mincle in 3 mL was performed with 40 units of furin at 37 °C for 18 h.

### Analytical procedures

SDS-polyacrylamide gel electrophoresis was performed on gels containing 17.5% acrylamide according to Laemmli (1970), followed by Coomassie blue staining or western blotting onto nitrocellulose. Blots were blocked in 5% bovine serum albumin in Tris-buffered saline, pH 7.8 for 30 min at room temperature, followed by overnight incubation with Mouse monoclonal anti-CLECSF9 antibody (Abnova) at 1:10,000 dilution to detect mincle followed by goat anti-mouse IgG/IgM antibody conjugated to alkaline phosphatase (Jackson ImmunoResearch Laboratories) at 1:5000 dilution (Merck). For detecting FcRγ, rabbit polyclonal anti-FcRγ antibody (Millipore) was used at 1:2000 dilution as a primary antibody and protein A-conjugated to alkaline phosphatase was used at 1:5000 dilution as a secondary reagent.

Gel filtration analysis was performed on a 1 × 30 cm Superdex-S75 column (GE Healthcare Life Sciences) eluted with 10 mM Tris-Cl, pH 7.8, 100 mM NaCl and 2.5 mM EDTA at a flow rate of 0.5 mL/min.

## Data Availability

Data are included in the article.
